# Octylamine mediated growth of europium doped silver selenide nanoparticles as a superior electrode material for electrochemical applications

**DOI:** 10.1039/d5ra03245h

**Published:** 2025-05-23

**Authors:** Tapan Kumar Sarangi, Rashmita Panda, Bhagaban Kishan, Kusha Kumar Naik

**Affiliations:** a P.G. Department of Physics, Berhampur University Odisha 760007 India kkn.phy@buodisha.edu.in; b Jawaharlal Nehru University New Delhi India

## Abstract

This study explores the synthesis and characterization of europium-doped silver selenide (Eu–Ag_2_Se) nanoparticles along with their application in electrochemical studies. The nanoparticles are synthesized *via* a hydrothermal method using silver, selenide and europium as precursors in the environment of octylamine solution. Comprehensive structural, morphological, and functional analyses are performed using X-ray diffraction (XRD), field-effect scanning electron microscopy (FESEM), high-resolution transmission electron microscopy (HRTEM), and Fourier transform infrared spectroscopy (FTIR). Electrochemical performance *i.e.*, in supercapacitors and glucose sensors, is assessed through electrochemical experiments like cyclic voltammetry (CV), galvanostatic charge–discharge (GCD), chronoamperometry (CA) and electrochemical impedance spectroscopy (EIS). It has been observed that Europium doping significantly enhanced the specific capacitance, achieving 337.8 F g^−1^ at a current density of 0.14 A g^−1^, with an energy density of 8.4 W h kg^−1^ and a power density of 29.9 W kg^−1^. Additionally, the materials exhibited excellent cyclic stability, retaining 93% of their initial capacitance after 6000 cycles. Similarly, the sensitivity of the Eu–Ag_2_Se nanoparticles is calculated as 0.52 μA μM^−1^cm^−2^ in the linear range having good stability, selectivity and reproducibility. These results highlight the potential of Eu–Ag_2_Se nanoparticles as a promising candidate for next-generation energy storage systems and glucose sensing applications.

## Abstract 2

This study explores the synthesis and characterization of europium-doped silver selenide (Eu–Ag_2_Se) nanoparticles along with their application in electrochemical studies.

## Graphical abstract

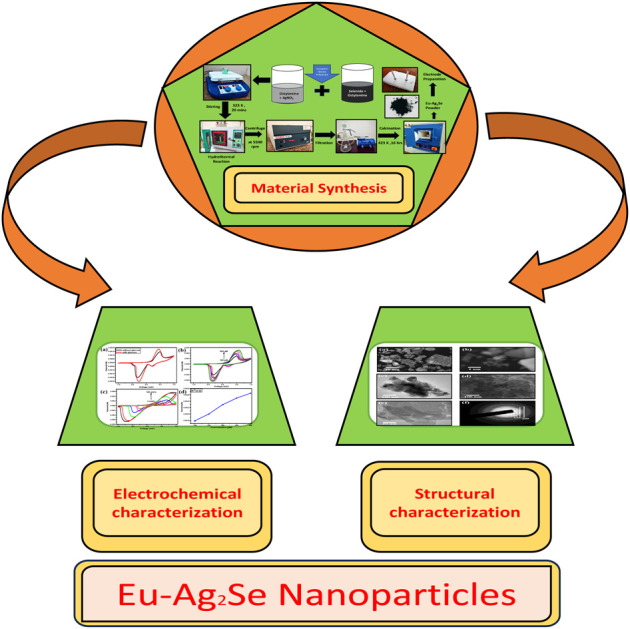

## 1 Introduction

The growing demand for renewable energy has increased the need for efficient energy storage systems. Supercapacitors stand out due to their high power density, rapid charge–discharge, and long-term stability. They bridge the gap between conventional capacitors and batteries by combining high power with moderate energy storage. Unlike batteries, which rely on slow electrochemical reactions, supercapacitors store energy through fast surface redox reactions. This allows ultrafast charging and discharging, making them ideal for applications requiring instant power delivery.^1–4^

Supercapacitors offer exceptional durability with a long cycle life, making them more cost-effective than batteries that degrade over time. They operate efficiently across a wide temperature range, ensuring reliability in extreme conditions. Their high power output makes them essential for renewable energy systems, electric vehicles, and industrial power backup.^5,6^ In hybrid and electric vehicles, they enhance efficiency by capturing and releasing energy during regenerative braking. Sustainability is a key focus, with researchers developing eco-friendly electrode materials like carbon nanostructures and transition metal oxides. Unlike conventional batteries, supercapacitors can be made from abundant, non-toxic, and recyclable materials, reducing environmental impact. They stabilize renewable energy sources by buffering fluctuations in solar and wind power generation. Additionally, they play a crucial role in smart grids, portable electronics, aerospace, and industrial applications. Ongoing advancements in material science and nanotechnology continue to improve their energy density and affordability. With superior power handling, long lifespan, and eco-friendly potential, supercapacitors are shaping the future of energy storage.^7–9^

Recently, non-enzymatic glucose sensors have attracted a lot of attention because of their ability to continuously monitor glucose levels, their high sensitivity, stability and their compatibility withbiological systems.^10^ Despite the prevalence of enzymatic test strips in glucose detection, the inherent instability of enzymes, attributed to their sensitivity to temperature fluctuations and pH variations, along with the necessity for mild operating and storage conditions, may compromise both the shelf life and sensing performance of glucose sensing strips. Consequently, it is imperative to create non-enzymatic glucose sensors as an alternative to mitigate some inherent drawbacks of enzymes, including pH- and temperature-dependent activity, and particularly the degradation of enzyme functionality in severe environments.^11^ A lot of research has been done on the creation of non-enzymatic glucose sensors using new materials that have unique nanostructures and compositions, A number of materials, including noble metals, metal oxides, carbon nanotubes, graphene, polymers, and composites, have been manipulated for their electrocatalytic reaction to the oxidation of glucose molecules.^12–15^

In recent years, the focus on electrode materials has become pivotal in enhancing electrochemical performances. Transition metal chalcogenides (TMCs) have garnered significant attention due to their unique combination of excellent electrical conductivity, thermochemical stability, and environmental compatibility.^6,16^ Among these, metal chalcogenides are getting more attention in research these days because they behave in different ways, are flexible, have a layer-like structure, are more conductive, work well as catalysts, have lower internal resistance, and have ohmic loss.^17–19^ Also, selenide is often used as an active electrode material because it has a higher conductivity (1 × 10^−3^ Sm^−1^) than sulfur (1 × 10^−28^ Sm^−1^). This means that electrochemical reactions happen faster on the electrode's surface and carriers move faster as well.^20,21^ Similarly, silver is the well-known transition metal for its conductivity and electrocatalytic nature. Because of these qualities, Ag_2_Se is the best transition metal selenide for electrodes: it has strong oxidation states, redox pairs, high electroconductivity, thermochemical stability, and safety in the environment.^22,23^ With Na_2_SO_4_ as the electrolyte, Shivasharma *et al.* looked at the orthorhombic structure of a thin film of silver selenide as an electrode material. A cyclic voltammetry test showed a specific capacitance of 112.4 F g^−1^ at a 10 mV s^−1^ scan rate, and a galvanostatic charge–discharge test showed a specific capacitance of 115.99 F g^−1^ at 0.8 A g^−1^.^24^ One more study by Shnag Wu *et al.* looked into the supercapacitive activity of NiSe/SnSe nanocomposite electrode material. They found that it had a specific capacitance of 116 mA h g^−1^.^25,26^

Metal and chalcogen based glucose sensors have attracted a lot of attention in recent years, due to the exceptional conducting properties and biocompatibility. Y. Tang, Q. Yang *et al.* designed a metal-enhanced QDs fluorescence system for the determination of glucose by conjugating CdSe QDs with AgNPs through the formation of reversible boronate ester bonds, which showed promising applications in chemical and biological sensors having a detection limit of 1.86 mM.^27^ A. D. Savariraj, V. Vinoth *et al.* designed a Three-dimensional (3D) bismuth selenide (Bi_2_Se_3_) nanostructure based non-enzymatic glucose sensor, which showed a low detection limit of 6.1 μM, a linear range from 10 μM to 100 μM of glucose concentration and a current sensitivity of 0.112 μAμM^−1^.^28^

To further improve the electrochemical performance of Ag_2_Se, material modification through doping has been explored extensively. Doping not only enhances the structural and electronic properties of materials but also introduces additional active sites for charge storage, thereby boosting overall capacitance.^16,29,30^ Rare-earth elements, such as europium (Eu), offer a distinct advantage in this regard. Europium's unique electronic configuration and redox flexibility can significantly enhance the conductivity and energy storage capability of silver selenide, making it a compelling choice for next-generation supercapacitor applications.^31–33^ Reviewing literature, pure and crystalline Europium doped silver selenide could be synthesized in an economical process. The synthesis parameters not only determine the structural and morphological characteristics but also deliver material properties like supercapacitive charge storage.^34^ Therefore, we could use an easy hydrothermal method to create Europium doped silver selenide nanoparticles, with octylamine facilitating the process. This would lead to enormous capacitance.^35–37^

In this study, europium-doped silver selenide (Eu–Ag_2_Se) nanoparticles were synthesized using a simple, economical hydrothermal method, with octylamine serving as a facilitating medium. The synthesis parameters were optimized to ensure a crystalline monoclinic lattice structure and desirable morphological characteristics. Characterization techniques, including X-ray diffraction (XRD), field-emission scanning electron microscopy (FESEM), high-resolution transmission electron microscopy (HRTEM), and Fourier-transform infrared spectroscopy (FTIR), were employed to confirm the structural and optical properties of the synthesized material.

This study presents the first report on the synthesis and dual-functional applications of europium-doped silver selenide (Eu–Ag_2_Se) nanoparticles *via* a simple, economical hydrothermal method using octylamine as a facilitating agent. The novelty lies in the strategic incorporation of europium, a rare-earth element, into the Ag_2_Se lattice, which significantly enhances its electrochemical properties by introducing additional redox-active sites and improving electrical conductivity.^38,39^ It is expected that, the synthesized Eu–Ag_2_Se would exhibit enhanced electrochemical characteristics like energy storage and bio-sensing applications. Unlike previously reported transition metal chalcogenide-based materials, the Eu–Ag_2_Se nanostructures demonstrated outstanding supercapacitive behavior, with a specific capacitance of 337.8 F g^−1^, and exceptional performance as a non-enzymatic glucose sensor, achieving a high sensitivity of 0.52 μA μM^−1^cm^−2^, detection limit of 60 μM, and fast response time 10 s. Additionally, the material exhibited excellent selectivity, reproducibility ±2% error, and stability in physiological conditions, highlighting its dual potential in both energy storage and biosensing, a unique combination rarely reported in literature. This dual functionality, enabled by a facile and scalable synthesis route, offers a promising pathway for the development of cost-effective, multifunctional electrode materials for next-generation energy and biomedical applications.

## 2 Experiment and measurement

### 2.1. Material synthesis

The growth mechanism of europium-doped silver selenide (Eu–Ag_2_Se) nanoparticles synthesized *via* a simple hydrothermal method involves a series of coordinated chemical transformations and physical processes. At first, 0.2717 g (80 mM) of AgNO_3_ was mixed with 10 ml of octylamine and, 0.0256 g (40 mM) of selenide powder was also mixed with 10 ml of octylamine in separate beakers and both of these mixtures were stirred for 20 minutes. The basic medium helps stabilize the ions and promotes controlled reaction kinetics. Europium nitrate trihydrate is introduced at a 3 mol% concentration in the disperse solution. Upon mixing the solutions and subjecting them to hydrothermal treatment at 423 K for 16 hours in a sealed autoclave, thermal energy drives the redox interaction between Ag^+^ and Se^2−^, resulting in the *in situ* nucleation and growth of Ag_2_Se nanoparticles through the reaction:2Ag^+^ + Se^2−^ → Ag_2_Se

**Fig. 1:**
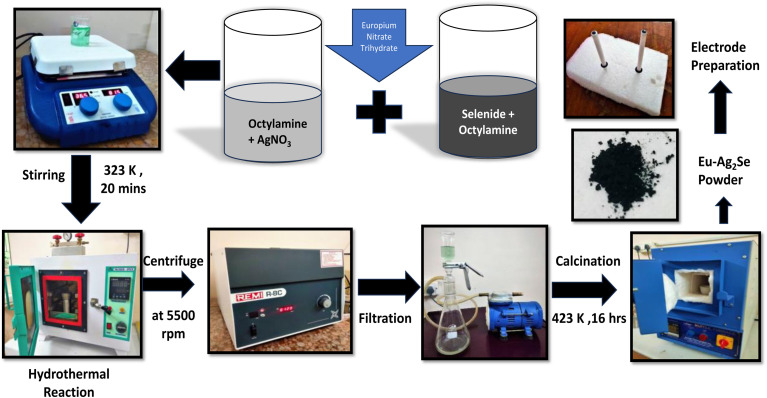
Schematic representation of synthesis process of the Eu–Ag_2_Se nanoparticles.

Europium ions (Eu^3+^) are incorporated into the Ag_2_Se crystal lattice by partially substituting Ag^+^ sites or occupying interstitial positions, depending on the reaction kinetics and thermodynamics. Octylamine assists in regulating this growth process by preventing agglomeration and directing morphology due to its long-chain structure and ability to bind with the nanoparticle surface. This doping introduces structural defects and additional electroactive sites, significantly improving the material's electrical conductivity and charge storage capabilities. The hydrothermal environment supports the uniform integration of europium, facilitating a stable crystalline phase. As the temperature-induced reaction proceeds, the nanoparticles further crystallize into a monoclinic structure, influenced by europium's presence and synthesis parameters. Upon cooling, the former Eu–Ag_2_Se nanoparticles precipitate out and are collected for washing, drying, and further characterization. Fig. 1 depicts the synthesis procedure of the synthesized Eu–Ag_2_Se material to enhance understanding and visualization of the process.

### 2.2. Material characterization

The crystal structure of the synthesized material is determined using an X-ray diffractometer (PROTO AXRD) with Cu Kα radiation (*λ* = 1.54 Å). The morphological features of the material is analysed through field emission scanning electron microscopy (FESEM, Zeiss Pvt. Ltd, Germany) and transmission electron microscopy (TEM, JOEL, 2100F). Functional groups associated with the synthesized material is identified using a Fourier-transform infrared (FT-IR) spectrometer (Bruker, ALPHA II).

### 2.3. Electrochemical measurement

Electrochemical measurements for the supercapacitor study were performed using a potentiostat (Sinsil International Ltd) in a three-electrode configuration with 1 M KOH as the electrolyte. The working electrode comprised synthesized europium doped silver selenide (Eu–Ag_2_Se) pasted with Nafion on a glassy carbon electrode. A platinum wire served as the counter electrode, and a saturated calomel electrode (SCE) was employed as the reference electrode. Cyclic voltammetry (CV) experiments were conducted within a potential window of −0.3 V to 0.3 V at varying scan rates to identify the redox potential of the Eu–Ag_2_Se material. Galvanostatic charge–discharge (GCD) tests were carried out at different current densities over the same voltage range to evaluate the charge-storage performance. Electrochemical impedance spectroscopy (EIS) measurements were performed in the frequency range of 10 kHz to 0.01 Hz to investigate the electrochemical characteristics of the electrode material. Using cyclic voltammetry and galvanostatic charge–discharge plots, the specific capacitance of Europium doped silver selenide material was calculated using eqn (1) and (2), as follows:1
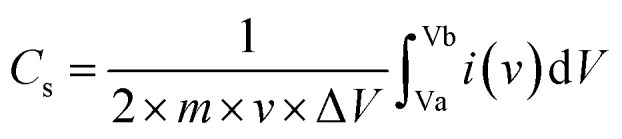
2
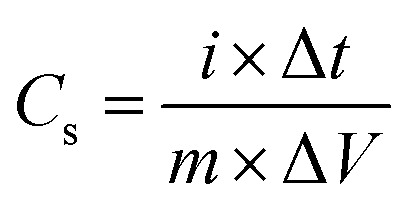
where ‘*i*’ is the current in (A), ʻ*m*’ is the mass of the electrode material in (0.1 mg), ‘Δ*t*’ is the discharge time in (s), scan rate is in (mV s^−1^), and potential window is in (V).

Cyclic voltammetry, chronoamperometry, and Interference studies were conducted using three electrode configurations and 0.1 M NaOH electrolytic solution to assess the electrochemical sensing capabilities of the Eu–Ag_2_Se nanoparticles. The material's electrochemical oxidation and reduction rates at the electrode surface were analyzed by CV tests conducted within a specified potential window.

The sensitivity was evaluated from the slope of the calibration curve, which is generally the plot between the measured output signal and analyte concentration. The formula that we have used is as follows:
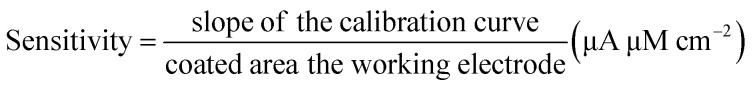


## 3 Result and discussion

### 3.1. Structural investigation

**Fig. 2:**
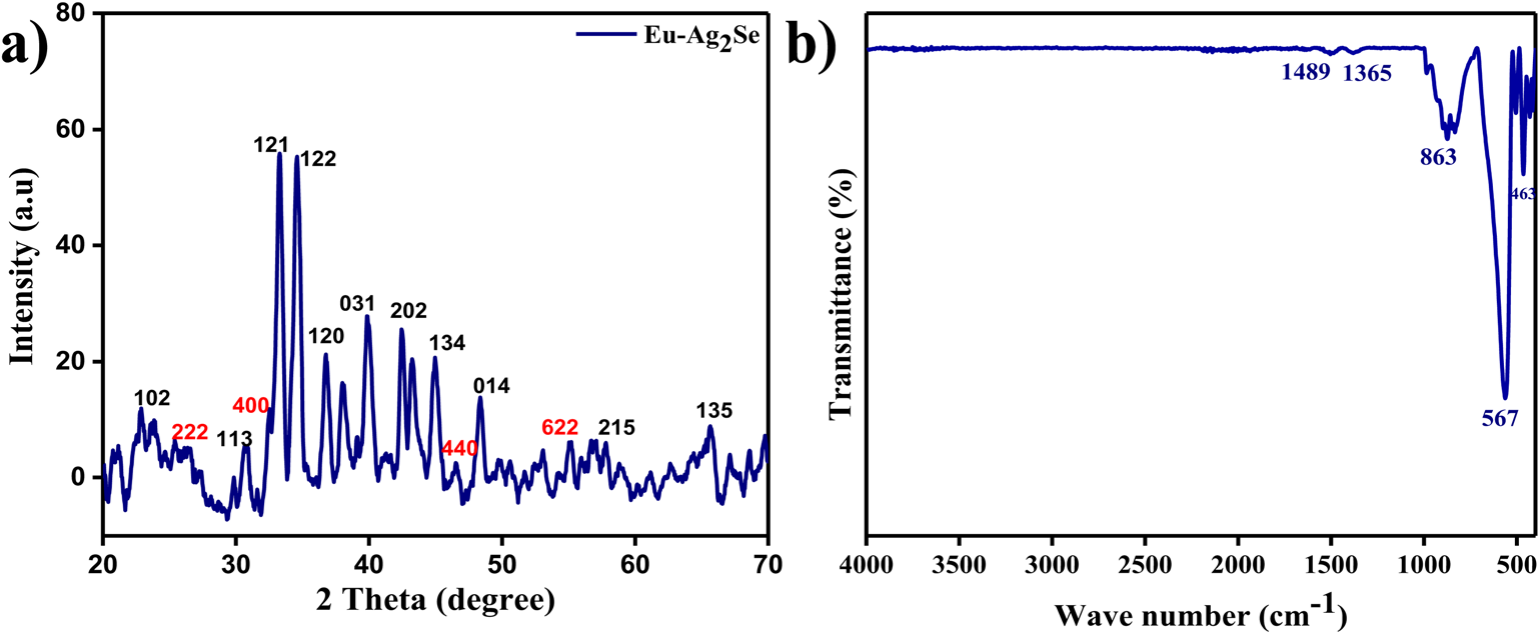
(a) XRD pattern of Eu–Ag_2_Se nanoparticles (b) FTIR spectrum of Eu–Ag_2_Se nanoparticles.

The structural properties of the Eu–Ag_2_Se material was analyzed using X-ray diffraction (XRD), and the corresponding diffracted pattern is presented in Fig. 2(a). The XRD pattern of the Eu–Ag_2_Se material displayed sharp, distinct, and well-resolved peaks at specific 2*θ* angles, corresponding to the Miller indices (−102), (−222), (−400), (−113), (−121), (−122), (−120), (−031), (−202), (−134), (−440), (−014), (−215), (−622), and (−135), respectively. The pronounced intensity of these peaks indicates the high crystallinity of the material. Among these, the peaks corresponding to the (−222), (−400), (−440), and (−622) planes are particularly significant, as they confirm the successful doping of europium into the Ag_2_Se lattice. The remaining peaks are attributed to the pristine Ag_2_Se phase, further validating the structural integrity and phase purity of the synthesized material. All observed peaks were indexed to the Eu–Ag_2_Se phase, consistent with the standard JCPDS card (01-075-1061). The absence of any additional peaks confirmed the phase purity and high crystalline quality of the synthesized Eu–Ag_2_Se material. The FT-IR spectrum of the synthesized Eu–Ag_2_Se material, presented in Fig. 2(b), confirmed the presence of oxygen-containing functional groups in the composition. Characteristic peaks were observed at 1489 cm^−1^, 1365 cm^−1^, 863 cm^−1^, 567 cm^−1^, and 463 cm^−1^, corresponding to the vibrations of C=C, C–H, Se–O, Eu–Se, and Ag–Se groups, respectively. These peaks confirmed the successful synthesis and composition of the Eu–Ag_2_Se material.

**Fig. 3:**
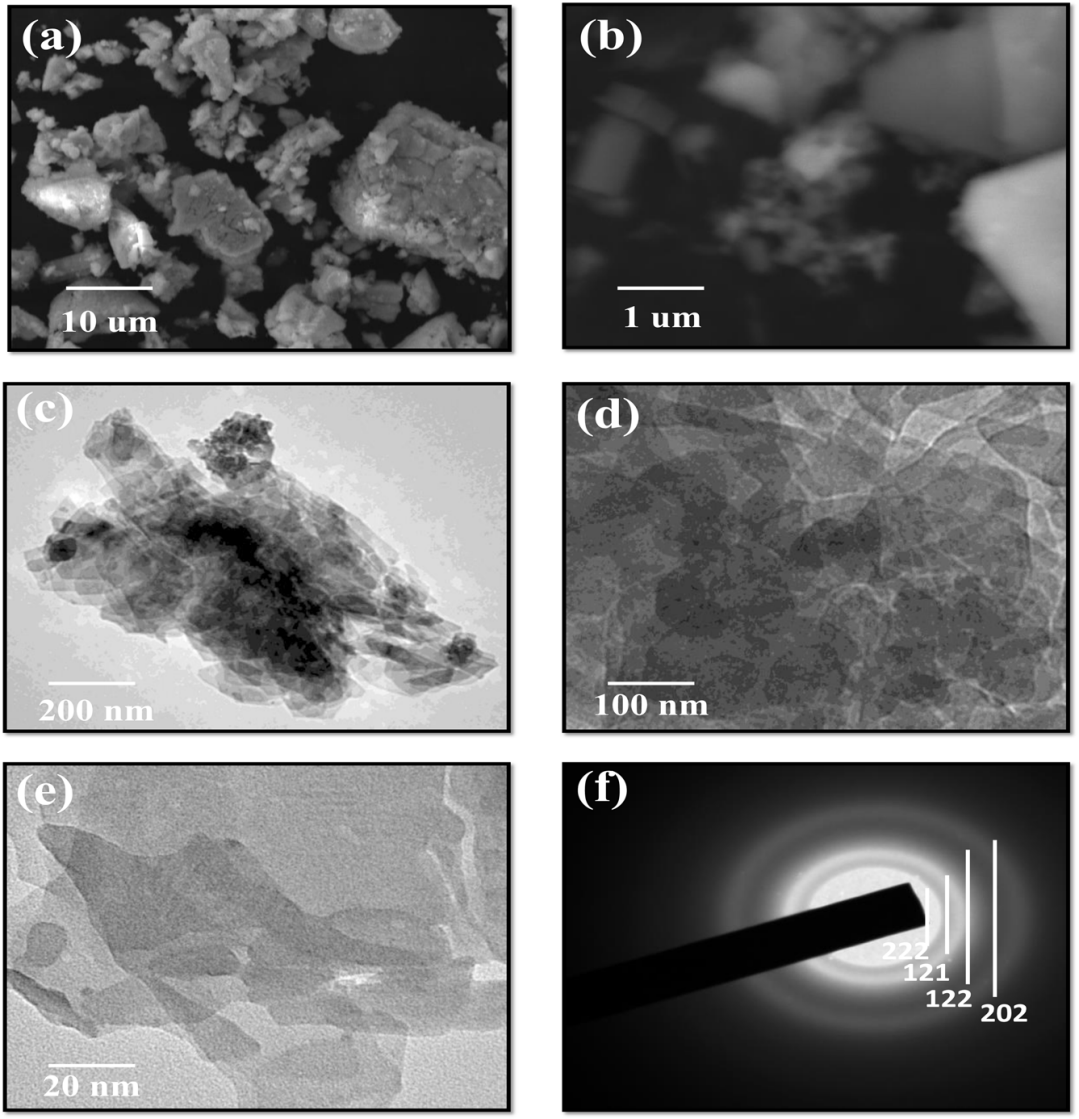
(a and b) Low and high resolution FESEM images, (c and d) TEM images at 200 nm and 100 nm, (e and f) HRTEM image and SAED pattern of Eu–Ag_2_Se nanoparticles.

The field emission scanning electron microscopy (FESEM) images in Fig. 3(a) and (b) reveal the morphological characteristics of Eu–Ag_2_Se nanoparticles. The low-resolution FESEM image (a) displays an aggregation of irregularly shaped nanoparticles with a broad size distribution, indicating a polycrystalline nature. This may be attributed to the introduction of europium ions, which disrupt the typical growth behavior of pure Ag_2_Se by altering the nucleation kinetics and promoting the formation of heterogeneous shapes. The high-resolution FESEM image (b) provides a closer view, highlighting the fine structural details and the presence of relatively larger crystallites interspaced with smaller grains. These structural distortions and boundary variations, resulting from Eu^3+^ incorporation, could enhance the surface area and increase the number of electroactive sites which is an advantage for electrochemical applications. Moreover, the variation in particle morphology and texture implies improved charge transport pathways and ion accessibility, both of which are pivotal for efficient energy storage. Thus, the FESEM observations affirm that europium doping not only modifies the physical structure of Ag_2_Se nanoparticles but also positively influences their electrochemical functionality of the material.

The transmission electron microscopy (TEM) and high-resolution TEM (HRTEM) images in Fig. 3(c–e) further illustrate the nanostructure and lattice arrangements of the synthesized Eu–Ag_2_Se nanoparticles. The TEM images (c and d) confirm the presence of thin, stacked nanosheets and agglomerated clusters, with varying levels of transparency indicating different thicknesses. The HRTEM image (e) showcases well-defined lattice fringes, signifying the crystallinity of the sample. Additionally, the selected area electron diffraction (SAED) pattern (f) exhibits distinct diffraction rings corresponding to indexed crystal planes (222, 121, 122, and 202), confirming the polycrystalline nature of the Eu–Ag_2_Se nanoparticles.

### 3.2. Supercapacitor study

The electrochemical storage performance of the Eu–Ag_2_Se nanoparticles are determined by performing a CV and GCD experiment taking three electrode configurations set up and 1 M of KOH electrolytic solution. At first the potential window of the material was finalized by performing the CV at a scan rate of 20 mV s^−1^, then CVs of different rates were executed successively.

**Fig. 4:**
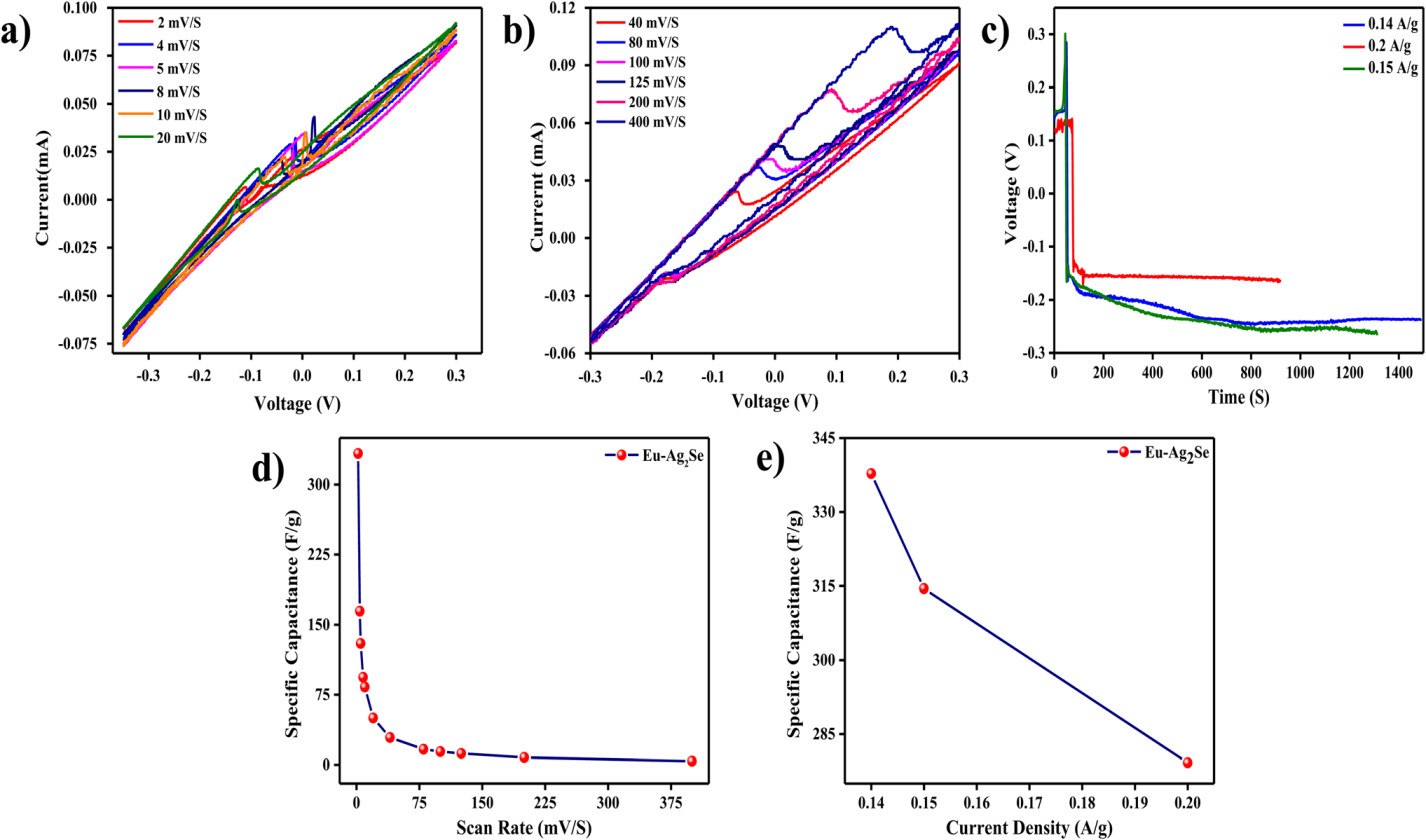
(a and b) CV curves of Eu–Ag_2_Se nanoparticles at different scan rates (2–400) mV S^−1^, (c) GCD curves of Eu–Ag_2_Se nanoparticles at different current densities (d) plot between specific capacitances with various scan rates (e) plot between specific capacitances with various current densities.

The CV curves in Fig. 4 (a and b) represented the electrochemical performance of the Eu–Ag_2_Se nanoparticles, recorded at scan rates ranging from 2 mV s^−1^ to 400 mV s^−1^. The shape of the curves exhibited a near-quasi-rectangular geometry, especially at lower scan rates, indicating typical capacitive behaviour. The consistent shape at varying scan rates suggested a combination of electric double-layer capacitance (EDLC) and faradaic Pseudocapacitance contributions.

At lower scan rates, the broader area under the curve reflected better utilization of the active sites due to sufficient time for ion diffusion into the electrode material. However, as the scan rate increased, the curves showed slight distortion and narrowing, especially at the peaks, due to kinetic limitations of ion transport. This limited area implies insufficient ion diffusion into the electrode material, potentially due to low porosity, poor conductivity, and inhomogeneous surface structure. Additionally, the absence of prominent redox peaks at higher scan rates, where faradaic reactions typically become more visible, could reflect incomplete or inefficient electrochemical activity possibly due to irregular europium and structural defects that hinder ions transport.

At scan rates above 200 mV s^−1^, a deviation from the ideal rectangular shape became noticeable. This behavior suggested that, at high rates, the charge storage process became surface-controlled, as ions failed to penetrate the bulk of the material efficiently. The symmetry of the CV curves, even at high scan rates, indicated low resistive losses and good electrochemical reversibility of the electrode. The ability of the Eu–Ag_2_Se nanoparticles to maintain capacitive characteristics at such high scan rates demonstrated the material's potential for applications requiring fast charge/discharge cycles, such as energy storage in power devices.

The GCD curves in Fig. 4(c) exhibited symmetrical triangular shapes across various current densities, confirming excellent electrochemical reversibility and a low equivalent series resistance (ESR) for the Eu–Ag_2_Se electrodes. At lower current densities, the discharge time was longer, reflecting higher specific capacitance due to better utilization of the active sites and a more thorough redox process. As the current density increased, the charge–discharge times decreased significantly. This reduction indicated that, at high current densities, the rapid charge/discharge cycles limited the penetration of ions into the bulk of the electrode material, leading to incomplete utilization of active sites. The nearly linear slopes of the charge and discharge curves also pointed to highly capacitive behavior with minimal energy losses during the process. A sudden voltage drop (IR drop) at the start of the discharge curves, which is characteristic of high internal resistance and poor coulombic efficiency. This phenomenon often arises from poor contact between the electrode material and current collector, non-uniform material distribution, and inadequate binding of the active material, leading to loss of electrical connectivity.

The plot in Fig. 4(d) showed the variation of specific capacitance as a function of scan rates. At lower scan rates, the specific capacitance was significantly higher, indicating better utilization of the electrode's active material. Slower scan rates provided ample time for electrolyte ions to diffuse and interact with the bulk of the electrode material. However, as the scan rate increased, the specific capacitance decreased due to the dominance of surface-controlled charge storage processes. At high scan rates, only the outermost active sites of the material participated in the charge–discharge process, while the inner sites remained largely unutilized due to diffusion limitations. This trend was typical for capacitive materials and highlighted the trade-off between energy and power density.

Table 1 displayed the calculated specific capacitance of Eu–Ag_2_Se nanoparticles using eqn (1) from the CV curve.

**Table 1:** The specific capacitance of Eu–Ag_2_Se nanoparticles at different scan rates

Scan rate (mV s^−1^)	2	4	5	8	10	20	40	80	100	125	200	400
Specific capacitance (F g^−1^)	333.3	164.5	130	93.7	83.3	50	29.1	16.6	14.1	12	7.9	3.9

Fig. 4(e) depicted the dependence of specific capacitance on current density. At lower current densities, the specific capacitance reached its maximum, indicating efficient charge storage facilitated by thorough ion diffusion into the electrode's pores. However, as the current density increased, the specific capacitance decreased significantly. The reduction in capacitance at higher current densities was attributed to the insufficient time for ions to interact with the deeper active sites of the electrode material. This limitation resulted in reduced charge storage capacity at higher power outputs. Despite this decrease, the Eu–Ag_2_Se nanoparticles demonstrated reasonable capacitance values even at high current densities, emphasizing their applicability in fast-charging energy storage devices. The specific capacitance of Eu–Ag_2_Se nanoparticles is calculated using eqn (2) from and the results are given in Table 2.

**Table 2:** The specific capacitances of Eu–Ag_2_Se nanoparticles at different current densities

Current densities (A g^−1^)	0.14	0.15	0.2
Specific capacitance (F g^−1^)	337.8	314.49	279.2

**Fig. 5:**
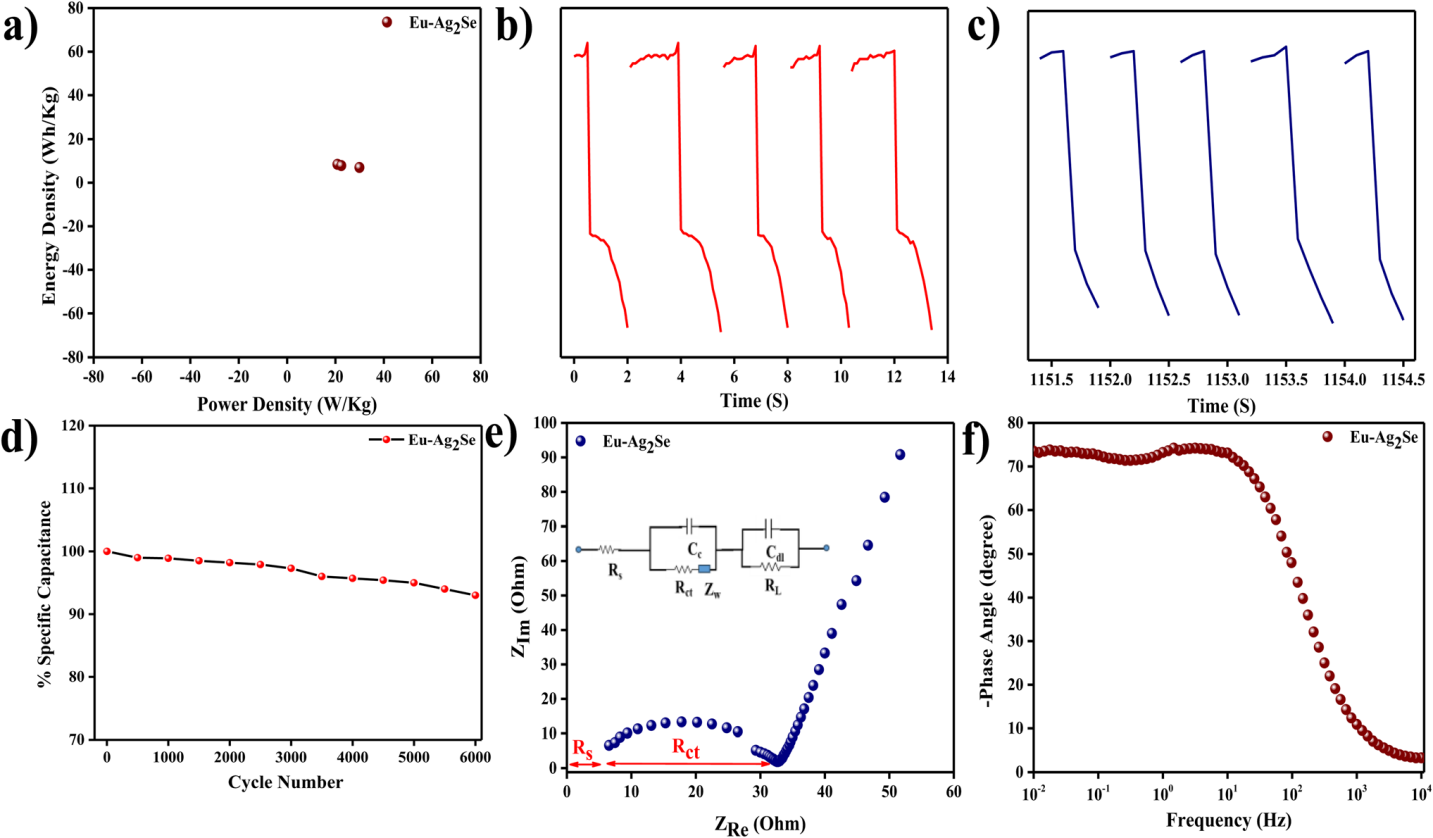
(a) Plot between energy density with power density, (b) charge and discharge time of Eu–Ag_2_Se of first five cycles, (c) charge and discharge time of Eu–Ag_2_Se of last five cycles, (d) plot between specific capacitances with various cycle numbers, (e) Nyquist plot of Eu–Ag_2_Se, (f) Bode plot of Eu–Ag_2_Se at frequency range 0.01 Hz to 10 kHz.

The Ragone plot in Fig. 5(a) illustrated the variation of energy density with power density for the Eu–Ag_2_Se electrode. The energy density decreased with an increase in power density, which was a typical trend for electrochemical energy storage devices. At lower power densities, the electrode delivered higher energy density due to the complete utilization of the electrode material and thorough ion diffusion. As the power density increased, the rapid charge–discharge process limited the storage capability, leading to a reduced energy density. This trade-off highlighted the material's ability to balance energy and power density, making it suitable for applications requiring both quick energy delivery and substantial energy storage.

Fig. 5(b) showed the charge and discharge profiles of Eu–Ag_2_Se nanoparticles during the initial five cycles. The near-symmetrical nature of the curves suggested excellent electrochemical reversibility and stability of the electrode material in the early stages. The consistent time intervals for charging and discharging implied minimal resistive losses and efficient energy storage. Additionally, the slight increase in discharge time over the first few cycles indicated an activation process, where the active sites of the electrode material became fully utilized.

Fig. 5(c) presented the charge and discharge profiles during the last five cycles. A comparison with the initial cycles revealed a noticeable decrease in the discharge time, indicating some degradation of the electrode material or partial loss of active sites over prolonged cycling. The symmetrical shape of the charge–discharge curves was retained, which confirmed that the material maintained its capacitive behavior, albeit with a reduction in storage capacity. This decline highlighted the need for further optimization to enhance the long-term cycling stability of the electrode.

The plot in Fig. 5(d) depicted the variation of specific capacitance with the number of cycles during cycling stability testing. The specific capacitance gradually decreased with increasing cycle numbers, reflecting some degradation of the electrode material over time. The retention of a significant portion of the initial capacitance after a large number of cycles indicated decent cycling stability of 93%, which was essential for practical supercapacitor applications. However, the observed decrease suggested that the electrode material might have been prone to structural changes or side reactions during prolonged operation.

The Nyquist plot in Fig. 5(e) displayed the impedance characteristics of the Eu–Ag_2_Se electrode. The presence of a small semicircle in the high-frequency region corresponded to the charge transfer resistance (*R*_ct_), indicating efficient electron transfer at the electrode–electrolyte interface. The linear portion in the low-frequency region represented the Warburg impedance, which was associated with ion diffusion within the electrode. The relatively low *R*_s_ (surface resistance) and *R*_ct_ values (3 ohm and 35 ohm) indicated excellent electrical conductivity and low resistance for ion transport, which were crucial for high-performance energy storage.

The Bode plot in Fig. 5(f) provided insight into the frequency-dependent behavior of the Eu–Ag_2_Se electrode. At lower frequencies, the phase angle approached 90°, indicating capacitive behavior dominated by ion diffusion and charge storage. At higher frequencies, the phase angle decreased, reflecting a transition to resistive behavior as the response time of the system became limited. The frequency ranges from 0.01 Hz to 10 kHz demonstrated the material's capability to operate across a wide spectrum, which was desirable for energy storage devices with varying operational requirements. Table 3 shows the comparison of specific capacitances of Eu–Ag_2_Se nanoparticles with other selenide materials.

**Table 3:** Comparison table of specific capacitances of Eu–Ag_2_Se nanoparticles with other selenide materials

Composition	Techniques	Specific capacitance (F g^−1^)	Reference
NiSe–Se	CVD	271.9	40
NiSe/SnSe_2_	Hydrothermal	116	25
Ag_2_Se	SILAR	115.9	24
MoSe_2_/rGO	Hydrothermal	211	41
NiSe_2_	Hydrothermal	75	42
CuSe	Solid state	209	26
Eu–Ag_2_Se	Hydrothermal	337.8	Present work

### 3.3. Glucose sensor study

**Fig. 6:**
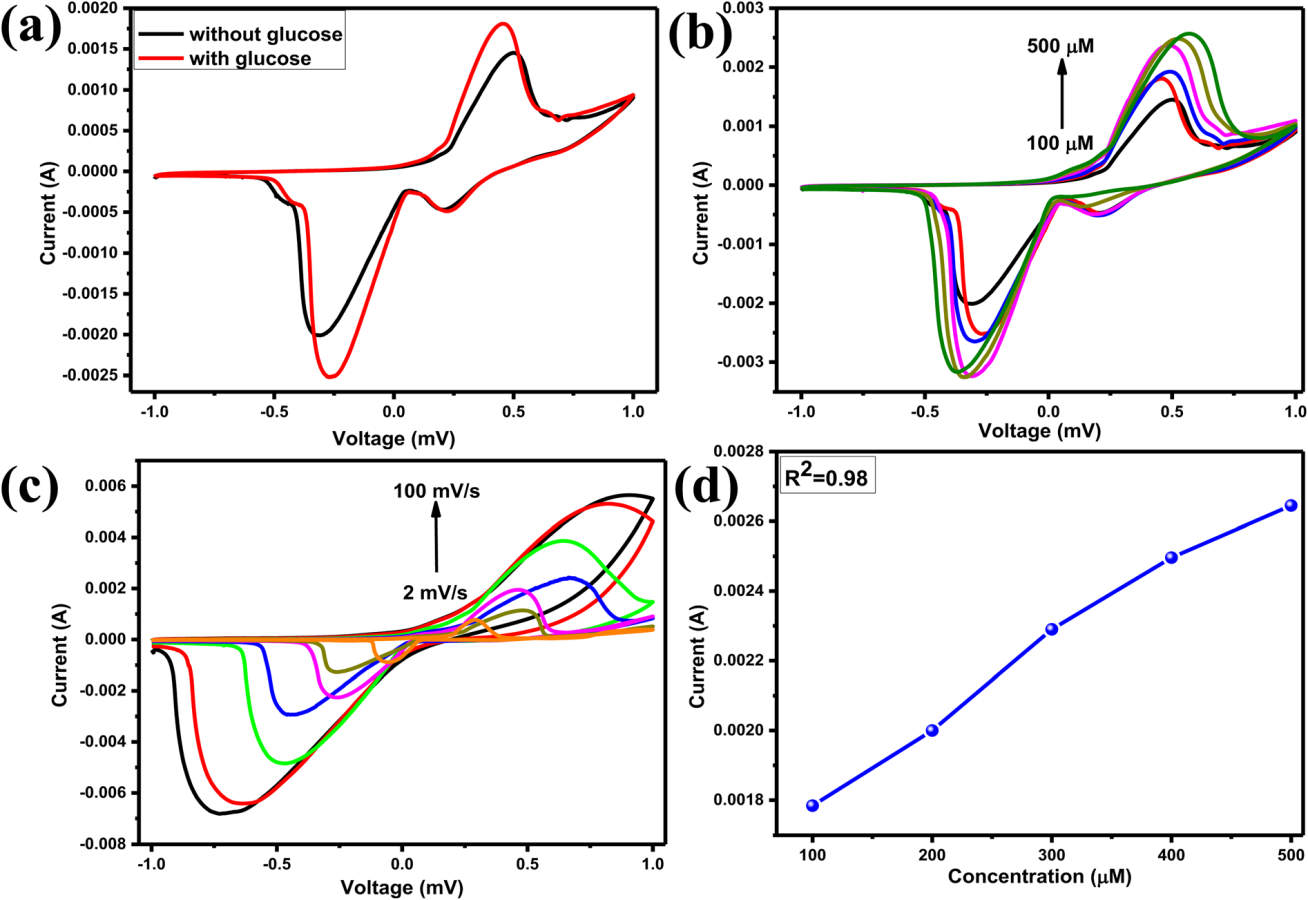
(a) CV of the Eu–Ag_2_Se nanoparticles without and with glucose concentration performed in the presence of 0.1 M of NaOH electrolytic solution, (b) CV of the Eu–Ag_2_Se nanoparticles at different concentration of glucose molecules, (c) CV of the Eu–Ag_2_Se nanoparticles at different scan rates and (d) graph between oxidation peak current with their correspondence glucose concentration.

The CV tests were conducted using 10 ml of 0.1 M NaOH electrolytic solution as the supporting electrolyte. As seen in Fig. 6(b), the redox peak current has increased with each additional quantity of glucose. This is because more and more electrons are released as the concentration of glucose increases. Fig. 6(d) demonstrates the linearity of the oxidation peak current, which indicates that the synthesized material has favorable catalytic characteristics in relation to the rising concentration of glucose. Fig. 6(c) shows the results of the CV of different scan rates, which proves that diffusion and kinetic coefficients are uniform and homogenous.

**Fig. 7:**
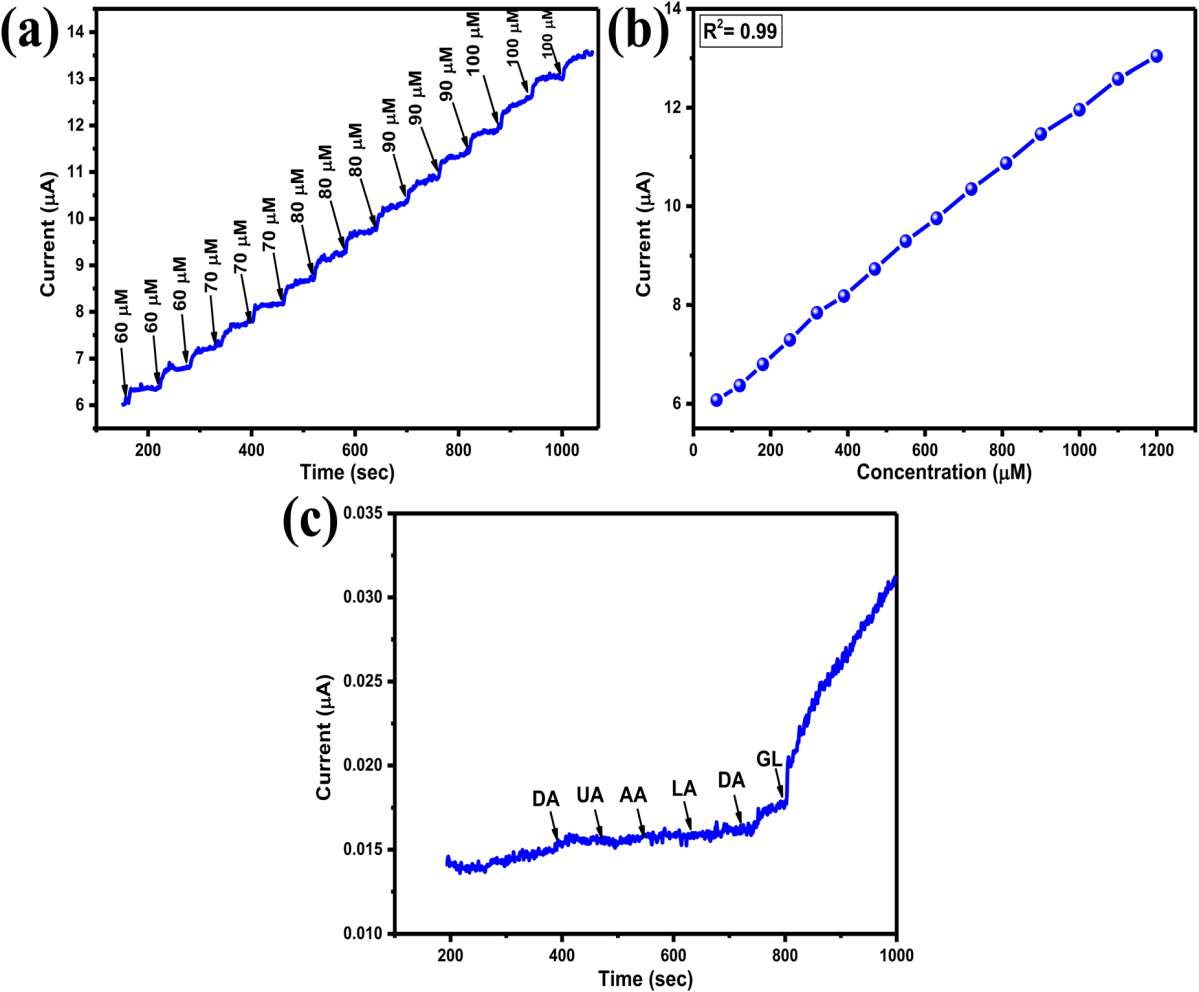
(a) IT curve of Eu–Ag_2_Se nanoparticles (b) calibration curve of Eu–Ag_2_Se nanoparticles. (c) Interference study of Eu–Ag_2_Se nanoparticles.

The amperometry response of Eu–Ag_2_Se nanoparticles was examined using 140 ml of NaOH solution in a glass cell, with a potential of 0.35 V applied to the three electrodes to observe the staircase-like current response of the synthesized materials. Upon the introduction of 60 μM glucose concentration into the solution, a sudden increase in current value was noted, followed by a horizontal saturation. Subsequently, varying amounts of glucose concentrations were added successively at 60-seconds intervals, as illustrated in Fig. 7(a).

The calibration graphs were plotted taking the current value in each addition of glucose amount dropped in the solution. The calibration curve shows a linear nature and sensitivity of the Eu–Ag_2_Se nanoparticles were calculated to be 0.52 μA μM^−1^ cm^−2^.

A crucial characteristic of an effective biosensor is its high selectivity for glucose analytes amidst a matrix of other chemicals and biomolecules. The interference study was conducted at a potential of 0.35 V, similar to the chronoamperometric study. Initially, 0.05 M solutions of various interfering species, including ascorbic acid (AA), lactic acid (LA), dopamine (DA), and uric acid (UA), were prepared in separate 0.1 M NaOH solutions. Subsequently, these interfering species were introduced into the solution continuously at equal intervals, as illustrated in Fig. 7(c). The Eu–Ag_2_Se nanoparticles demonstrated significant selectivity for glucose molecules while exhibiting minimal catalytic activity towards additional biomolecules.

## 4 Conclusion

In this study, we have synthesized Europium doped silver selenide (Eu–Ag_2_Se) nanoparticles using a cost-effective hydrothermal method, employing octylamine as the growth medium. The material was thoroughly characterized *via* XRD, FE-SEM, TEM and FT-IR, confirming its crystalline structure and nanoparticle morphology. Its electrochemical properties were evaluated in a three-electrode setup with KOH as the electrolyte, achieved a specific capacitance of 337.8 F g^−1^ at a scan rate of 0.14 A g^−1^ with Energy density of 8.4 W h kg^−1^ and Power density of 29.9 W kg^−1^. Also retained 93% capacitance after 6000 cycles, demonstrating excellent cyclic stability. The sensitivity of the prepared material for glucose sensing is calculated to be 0.52 μA μM^−1^cm^−2^.

## Data availability

All data supporting this study has been included in the article.

## Conflicts of interest

There are no conflicts to declare.
